# Multifunctional Pharmaceutical Effects of the Antibiotic Daptomycin

**DOI:** 10.1155/2019/8609218

**Published:** 2019-05-27

**Authors:** Yang Ye, Zijing Xia, Dan Zhang, Zenghua Sheng, Peng Zhang, Hongxia Zhu, Ningzhi Xu, Shufang Liang

**Affiliations:** ^1^State Key Laboratory of Biotherapy and Cancer Center, West China Hospital, Sichuan University and Collaborative Innovation Center for Biotherapy, No.17, 3rd Section of People's South Road, Chengdu 610041, China; ^2^Department of Nephrology, West China Hospital, Sichuan University, Chengdu, Sichuan 610041, China; ^3^Department of Urinary Surgery, West China Hospital, Sichuan University, Chengdu, Sichuan 610041, China; ^4^Laboratory of Cell and Molecular Biology & State Key Laboratory of Molecular Oncology, Cancer Institute & Cancer Hospital, Chinese Academy of Medical Sciences, Beijing 100034, China

## Abstract

Daptomycin (DAP), a cyclic lipopeptide produced by* Streptomyces roseosporus*, is a novel antibiotic to clinically treat various Gram-positive pathogenic bacteria-induced infections. Although DAP has a strong broad-spectrum bactericidal effect, recently rare bacterial antibiotic resistance against DAP gradually arises. The review is to summarize the normal indications of DAP, its off-label usage against several clinical pathogen infections, the unique antibacterial mechanisms of DAP, and the combination of antibiotic therapies for highly DAP-resistant pathogens. More noticeably, rising evidences demonstrate that DAP has new potential activity of anticancer and immunomodulatory effects. So far the multifunctional pharmaceutical effects of DAP deserve to be further explored for future clinical applications.

## 1. Introduction

*Streptomyces* are a rich source of bioactive secondary metabolites including numerous antibiotics. Among many antibacterial antibiotics, daptomycin (DAP), which is a secondary metabolite produced by* Streptomyces roseosporus*, is the first novel cyclic lipopeptide antibiotic (commercial name Cubicin®) approved clinically against various Gram-positive bacteria-induced infections [1].

DAP is an amphiphilic peptide with the molecular formula of C_72_H_101_N_17_O_26_, which is composed of 13 amino acids and a decanoyl fatty acid tail [2]. In particular DAP contains both hydrophilic and hydrophobic amino acids, which determine its amphiphilic characteristic. One special amino acid, kynurenine, is highly fluorescent, and its intrinsic fluorescence is available to detect the interaction of DAP with cell membranes by fluorescence confocal microscopy.

DAP has a strong kill activity against most of Gram-positive pathogenic bacteria. Because it is superior to vancomycin on the antimicrobial efficacy almost in all in vivo studies, DAP is commonly used as an effective therapy after vancomycin failure for the treatment of methicillin-resistant* Staphylococcus aureus* (MRSA) infections [3]. Beside its effective antimicrobial activity, latest study suggests that DAP is a new potential therapeutic with anticancer and immunomodulatory activity.

The aim of this review is to summarize the normal indications and off-label usage of DAP, its unique antibacterial mechanisms, the combination antibiotic therapies, and its treatment for DAP-nonsusceptible (DNS) pathogens. Further, the options to overcome drug-resistance are also discussed. It is specially noticed that DAP has great potential as a new therapeutic against cancer and other immune relative diseases.

## 2. Broad Antimicrobial Activity of DAP

### 2.1. Normal Indications of DAP

DAP (commercial name Cubicin®) is originally approved by Food and Drug Administration (FDA) for the main indications including complicated skin and skin structure infections in adult and pediatric patients,* Staphylococcus aureus (S. aureus)* bloodstream infections (bacteremia), and those with right-sided infective endocarditis in adult patients [2]. And, compared with linezolid and vancomycin, DAP is well tolerated and has been proven to be a successful and less toxic alternative to vancomycin for the treatment of drug-resistant Gram-positive pathogens [2]. Despite sporadic reports of DAP resistance emerge in recent years, DAP is still a very important antimicrobial for current clinical practice around the world.

Moreover, DAP has been enlarged to be applied for various bacterial infections, including off-label use for osteomyelitis, meningitis, febrile neutropenia, and left-sided endocarditis.

### 2.2. Off-Label Usage of DAP

DAP shows good efficacy in antibiotic-resistant strains including MRSA and vancomycin-resistant Enterococci (VRE), and DAP has been a preferred treatment strategy for VRE infections [4]. Furthermore, high dose of DAP is one alternative treatment recipe for penicillin-resistant and cephalosporin-resistant pneumococcal meningitis and MRSA osteomyelitis [5, 6]. In addition, DAP has rapid bactericidal effects on* Bacillus anthracis* by reducing membrane potential so that it can be used as an alternate therapy for* Bacillus anthracis* infections [7]. Generally, DAP is a valuable treatment method in the management of other Gram-positive and difficult-to-treat infections, including meningitis, osteomyelitis, febrile neutropenia, and left-sided endocarditis.

#### 2.2.1. Osteomyelitis

DAP is effective and safe in treatment for osteomyelitis [6]. Though it is not approved by FDA, the Infectious Diseases of Society of America guidelines for management of osteomyelitis recommend DAP as an alternative treatment method for osteomyelitis [8].

As DAP penetrates into synovial fluid and cancellous bone well, DAP concentrations in bone tissues are above the minimal inhibitory concentration (MIC) of DAP for* S. aureus* after a single-dose intravenous infusion [9]. Approximately 75% of cure rate is derived from the analysis of European Cubicin® Outcomes Registry and Experience registry data, with a low incidence of adverse events and drug resistance [10]. The optimal DAP dose to treat osteomyelitis has not been determined, although a study shows more than 6 mg/kg once daily is advantageous to compensate for low vascularization of bone tissue [8]. A high dose of 8-10 mg/kg DAP each day as monotherapy or in combination therapy with rifampin or trimethoprim-sulfamethoxazole optimizes its activity and avoids emergence of resistance [11].

#### 2.2.2. Meningitis

At present, DAP is not indicated for meningitis treatment as it does not easily enter the central nervous compartments [12]. But DAP concentration in the cerebrospinal fluid is as higher as more than 100 times of the MIC [5]. Moreover, due to its excellent bactericidal response with no lysis, DAP is applicable for useful alternative treatment with penicillin-resistant and cephalosporin-resistant pneumococcal meningitis at high doses like 25 mg/kg [5]. So far, the experience with DAP for meningitis treatment mostly is confined to case reports and needs a large number of clinical evaluations [13].

#### 2.2.3. Febrile Neutropenia

A few case reports have documented the success of DAP for Gram-positive infections in neutropenic patients [14, 15]. DAP appears to be well tolerated and successfully control superinfections of bacteremia without marrow toxicity and complications in the neutropenic population [16]. Notably, the potential role of DAP in febrile neutropenia is yet to be determined, and clinical investigations are ongoing including the neutrophil recovery, blood culture results, and inflammatory condition of the entry sites.

#### 2.2.4. Left-Sided Endocarditis

DAP was approved by FDA for the treatment of right-sided endocarditis in 2007, but its use for left-sided endocarditis therapy remains unapproved till now. Preliminary observation demonstrates good efficacy of DAP treatment in a small number of cases of left-sided infective endocarditis and MRSA-related left-sided endocarditis, where vancomycin is not effective [17, 18]. But a wider series of patients with left heart endocarditis are now recruited to confirm the efficiency of DAP therapy [19].

In addition, DAP is not indicated for use against pneumonia and respiratory disease because it is inhibited by pulmonary surfactant, although DAP shows good curative effect in a mouse model with hematogenous pulmonary infection induced by MRSA [20].

## 3. Antibacterial Mechanism of DAP

The bactericidal mechanism of DAP is different from other traditional antibiotics [21], such as linezolid, vancomycin, and dalbavancin, so there is no cross-resistance between DAP and these antibiotics. To date, several molecular mechanism models of DAP have not completely elucidated its authenticity.

The majority of studies support that DAP acts on cell membranes in the presence of calcium ions, substituting Ca^2+^ with any of these divalent cations such as Mn^2+^, Mg^2+^, Cu^2+^, and Ni^2+^, causing a minimum 32-fold increase in the MIC [22]. Once a 2:3 stoichiometric ratio of Ca^2+^ to DAP is reached, the conformation of DAP changes, which stimulates DAP oligomerization; finally its lipophilic tail inserts into bacterial cell membranes to penetrating membrane to induce loss of membrane potential and cell death [21, 23]. Our previous study has confirmed that the antibacterial mechanism of DAP is related to pathogen membrane proteins, including expression changes of multiple proteins [24]. The bacterial membrane potential is decreased and cell membrane is disrupted due to the upregulation of some proteins and downregulation of other proteins under DAP exposure, which contributes to rapid leakage of bacterial DNA and bactericidal effect [24]. While several pathogenic proteins are related to nucleic acid metabolism, DAP is probably involved in nucleic acid regulation. So the multitarget effects of DAP through proteomic dissection and validation are also worth considering[24, 25].

DAP seems to act on different targets for different infectious pathogens. DAP targets the fluid lipid domains, delocalizes different membrane proteins in* Bacillus subtilis*, and finally inhibits the synthesis of cell envelope by delocalizing different peripheral membrane proteins and suppressing the activity of biosynthetic enzymes [26]. DAP does not form discrete membrane pores in* Bacillus subtilis* [26, 27], which seems like self-contradictory with prevailing model about DAP ion channels. The differences in the fatty acyl composition of bacterial membranes could account for different susceptibilities toward membrane disruption [27]. The membrane pore formation is restricted by fatty acyl chain composition. Thus, the membrane pore is unable to form in DAP with cell membrane in* Bacillus subtilis *due to the bacterial membrane composition.

DAP exerts its antibacterial action not only on calcium ions but also with the presence of a high proportion of phosphatidylglycerol (PG) in membranes. The reduction of membrane lipids affects the affinity of DAP with membranes, which induces DAP inactivation by* S. aureus *[28]. Actually, DAP does not cross the giant unilamellar vesicles in the pathogen cell membranes [29, 30]. Generally, the antibacterial activity of DAP also depends on the membrane composition of various infectious bacteria.

## 4. New Potential Pharmaceutical Effects of DAP

### 4.1. DAP-Mediated Immunomodulatory Effect

**Figure 1:**
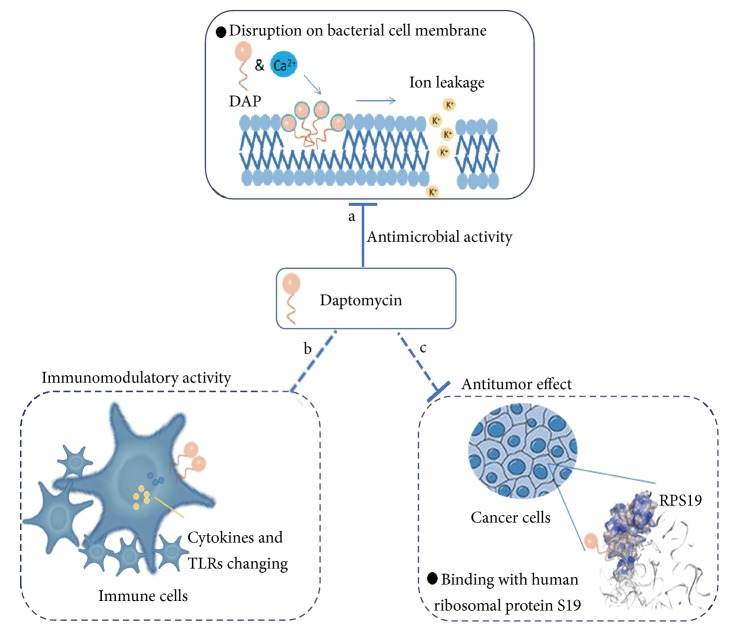
Multifunctional pharmaceutical effects of the antibiotic daptomycin. Besides antimicrobial properties, DAP is associated with anticancer and immunomodulatory activity.

Except own antimicrobial properties, DAP has been reported to exert other pharmaceutical effects, including immunomodulatory and anticancer activities (Figure 1).

In fact, lipopeptides interact with toll-like receptors to induce immune response [31]. DAP, a typical lipopeptide, may penetrate human monocytes to exert immunomodulatory effects [32, 33]. DAP also regulates host immune response through changing cytokine production [33]. DAP may suppress cytokine expression in the case of host immune response stimulation against MRSA [34]. For instance, in the inflammatory response during sepsis, DAP treatment induces a significantly higher mRNA level of IL-6, IL-10, and IL-1*β* downregulation in lipopolysaccharide-activated monocytes. DAP also downregulates the gene expression of TLR1, TLR2, TLR6, and TLR9, whereas it makes an upregulation of TLR4 and TLR7 [35]. DAP treatment significantly induces lower TNF-*α* levels in cerebrospinal fluid of animal models compared with ceftriaxone [36].

Because the PG component in cell membrane is much richer in bacteria than that in eukaryotic cells, DAP has less effect on eukaryotic cells but its accumulation in alveoli will cause antigenic stimulation and continuous immune activation [37]. This is reason to explain the poor efficacy of DAP to treat left-sided infective endocarditis compared with right-sided infective endocarditis [38]. To date, the interaction of DAP with normal eukaryotic cell membrane constituents has little been reported.

DAP attributes largely to the reduced presentation of bacterial components to the host, so it is essential to explore the synergistic potential with some immunomodulators to indirectly modulate host immune function during bacterial infection. For example, DAP shows synergistic effects in combination with vitamin E [33]. The treatment with vitamin E prior to infection is effective for improving efficacy of DAP in MRSA-caused wound infection [39]. This better combination effects have been validated in mouse models, in which CD49b+ cells are increased compared with single treatment with vitamin E or DAP alone [40]. Although the immunomodulatory mechanism of DAP is not completely explored, it is reasonable to predict DAP potential usage for immune-relative diseases. So DAP may have the potential to indirectly modulate host immune function during resolution of infection.

### 4.2. Antitumor Potential of DAP

Peptides have displayed anticancer properties [41]. Some marine cyclic peptides have anticancer applications, such as dolastatines and soblidotin, especially cyclic guineamides A-F which are similar to DAP in the molecular structures [41].

There are some preliminary investigations on DAP anticancer activities. DAP inhibits cell proliferation of cancer cells including MCF-7 and HCT116, and RPS19 is identified to be a biophysical and biologically related target protein of DAP through an unbiased reverse chemical proteomics screen [42]. With higher expression levels of RPS19 in certain colon cancer cells, RPS19 may be a promising drug target protein for DAP anticancer activity [42].

Gram-positive infections are a main cause of morbidity and mortality in cancer patients. Though clinical data for DAP in the treatment of cancer patients are limited, there are some evidences of DAP usage particularly in some special Gram-positive infections in cancer patients with neutropenia (Table 1) [43]. In addition, DAP is an attractive option for the treatment of Gram-positive catheter-related bloodstream infections in cancer patients [44] and implantable intra-arterial catheters in liver metastases of colon cancer [45].

Compared to other antibiotics used in cancer infections, in terms of efficacy and drug resistance, DAP is a safe and effective therapy in the treatment of bacterial infections induced by Gram-positive pathogens [46]. Both vancomycin and linezolid have limitations with respect to their use in patients with neutropenia. Compared with vancomycin, the nephrotoxicity of DAP is not significant; cancer patients treated with DAP achieve earlier microbiological eradication [47]. Furthermore, unlike linezolid, DAP is more effective in eradicating catheter-related MRSA infections [48].

An interesting question is whether the combination of DAP and antitumor drugs (e.g., oxaliplatin and letrozole) can enhance the activity of anticancer drugs. Because of the low immunity of cancer patients, perhaps DAP has synergistic antitumor effects for cancer patients in combination with anticancer drugs. However, this conjecture needs further study.

## 5. Overcoming Antibiotic Resistance

Gene mutations, changes in surface charges of cell membranes, the thickening of bacterial cell walls, and the altered lipid metabolism are all associated with DAP resistance in Gram-positive pathogens, including* Enterococcus faecalis *[49],* S. aureus* [28],* Streptococcus mitis/oralis* [50], and* Bacillus subtilis* [51]. Although this resistance is very sparse without wide spreading, the occurrence of DAP resistance during therapy seems to be an important problem affecting the clinical efficacy of DAP [28]. New DAP-based antibiotic discovery and combination therapy are considered to prevent the emergence of highly drug-resistant pathogenic bacteria [52].

### 5.1. DAP-Derived Antibiotics

Cyclic peptide antibiotics have unconventional antibacterial mechanisms due to their unique structure. Novel cyclic peptides have been derived from a natural product, fusaricidin/LI-F, to have better antibacterial activity through screening a positional scanning combinatorial library [53]. And, with the increasing of the overall hydrophobicity and the net positive charge of cyclic lipopeptides, the antibacterial activity will be improved. Besides, bicyclic peptides may be a good way to discover new cyclic bioactive peptides [54]. So de novo rational design and biosynthesis are applicable to optimize and create better therapeutic polypeptide-like drugs [52]. But, inconsistently, DAP dimer, which is composed of two subunits, has a very low activity on vegetative* S. aureus *because the dimer does not act on cell membranes due to the bivalent aliphatic acyl chain between two subunits [55].

In addition, enhancing membrane-binding property is an option to optimize antibiotics. For instance, N-terminal or C-terminal lipophilic groups (myristoyl, palmitoyl, geranyl, and farnesyl) of vancomycin are appending onto the C-terminus to enhance the membrane binding with antibiotic and drug concentration at the target site, which increases killing activity against MRSA and DAP-resistant bacteria [56]. The differences in the fatty acyl composition of bacterial membranes are related with DAP binding to the membranes. So, DAP activity is also influenced by the optimization of fatty acid side chains. For example, surotomycin, which is a DAP derivative, exhibits significant improvement of in vitro activity compared with DAP against* Clostridium difficile* [57]. The only change between them is the specific fatty acid side chains. Therefore, fatty acid side chains play an important role in the binding of antibiotics to cell membranes and affect antibacterial activity. More importantly, the optimal balance between strong antibacterial activity and optimum structure is essential for structure modification.

### 5.2. Synergism

A combination therapy of two antibiotics is recommended to prevent the emergency of DAP resistance. Herein, several combination antibiotic regimens are reported to be effective against DAP-resistant bacterial infections, and these combinations are summarized in Table 2.

#### 5.2.1. DAP Combined with *β*-Lactams

DAP combined with *β*-lactams, especially with ceftaroline and ampicillin, has better killing of DAP nonsusceptibility in clinical MRSA and enterococci isolates [58–60]. The affinity of DAP and bacterial membranes is found to be enhanced by the addition of ampicillin, which is proven to be the synergy mechanism between ampicillin and DAP in enterococci [59]. A combination of DAP and ceftaroline is active against DNS MRSA and* Streptococcus mitis* in vitro by enhancing the binding of DAP and cell membranes [61–63]. Interestingly,* S. aureus* inactivates DAP through releasing membrane phospholipids, which can be inhibited with the presence of *β*-lactam antibiotic [28]. DAP-oxacillin exposure reduces membrane amounts of penicillin-binding protein 2 and redirects the localization, which induces cell wall perturbations and increases susceptibility to oxacillin [58].

**Figure 2:**
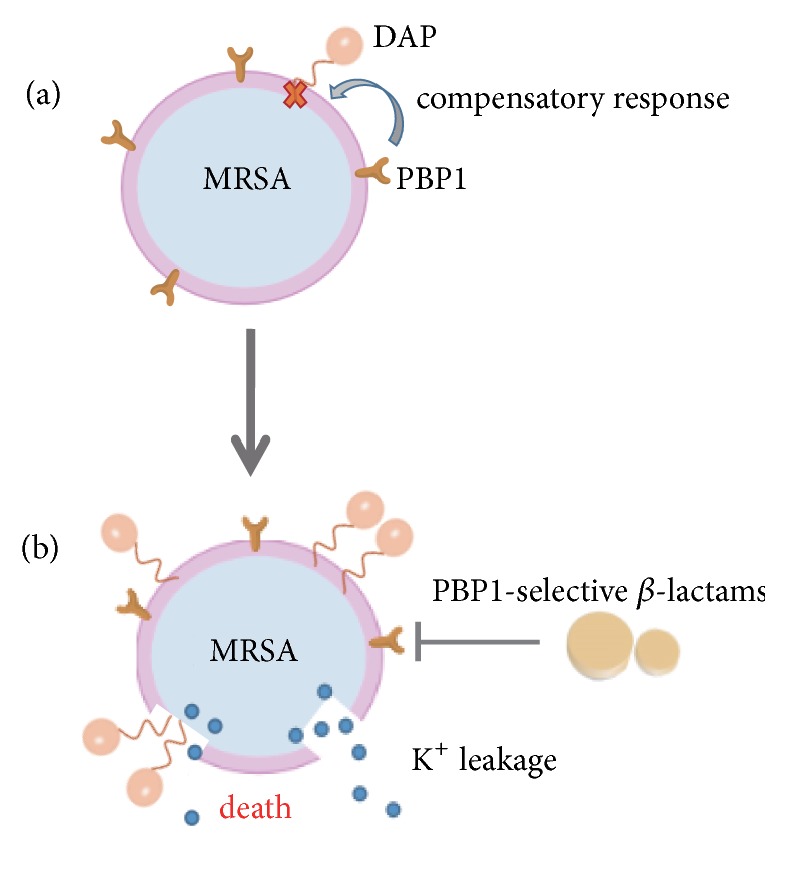
*The action mechanism of DAP combined with β-lactams*. (a) Penicillin binding protein 1 (PBP1) activity is related to DAP-induced metabolic stress, and it forms an adaptive response to DAP-mediated surface injury. (b) Targeting inactivation of PBP1 makes DAP-nonsusceptible* S. aureus* strains revert to subsequent DAP-susceptible strains, which enhances the binding of DAP with cell membrane and then induces cell death.

DAP/*β*-lactams have intracellular activity against MSSA and MRSA. Oxacillin binds to penicillin-binding proteins, which are enzymes involved in the synthesis of peptidoglycan. So oxacillin enhances the activity of DAP due to suppression of cell wall synthesis [64]. Meanwhile, penicillin binding protein 1 (PBP1) activity is associated with DAP-induced metabolic stress. PBP1 is one of penicillin binding proteins in* S. aureus*, which is a target of *β*-lactam. PBP1 is a crucial compensatory response to DAP injury. Targeting inactivation of PBP1 induces DNS* S. aureus* reversion to a subsequent DAP-susceptible strain, which alters the efficacy of DAP killing. Therefore DAP combined with PBP1-selective *β*-lactams may be a suitable strategy (Figure 2) [65]. In short, the synergistic bactericidal effects of DAP with other chemicals on the DNS strains are mostly dependent with the synergistic drug influence on membrane proteins, by facilitating an increase of DAP binding and rapidly leading to cell death.

#### 5.2.2. DAP Combined with Other Antibiotics

There are attempts for the synergy with DAP and other antibiotics, such as gentamicin [66], dalbavancin [67], and fosfomycin [68]. And the MIC values of DAP in combination with these antibiotics are lower than each alone [67, 68].

Fosfomycin is a phosphonic acid derivative, which is active against both Gram-negative and Gram-positive bacteria by inhibiting peptidoglycan synthesis. DAP influences peptidoglycan synthesis based on the above mechanisms. So fosfomycin can increase the activity of DAP against VRE through modulating cell surface charge, which indicates that the mechanism is similar to the combination of *β*-lactams and DAP [69]. Similarly, the combination of DAP plus gentamycin has demonstrated better activity against MRSA and also provides a promising synergy required to overcome DAP resistance in complicated infections [66]. Gentamicin is one of the most active aminoglycosides, which is used to treat bacterial infections, especially those caused by Gram-negative bacteria. It can bind to ribosomal 30s subunit and block bacterial protein synthesis, which is different from the action mechanism of DAP [66]. The synergistic mechanism is unclear, and the synergy is only demonstrated in in vitro model, so further study should focus on the synergy in vivo and clinical applications.

## 6. Future Prospects

Except for antimicrobial properties, DAP may have other pharmaceutical activities like immunomodulatory and antitumor effects. It still has a great development prospect to extend DAP clinical potentials. Firstly, extending the antimicrobial spectrum of DAP is pending because of the unapparent inhibition effect of DAP on Gram-negative bacteria. Recently, it has been reported that DAP has some influence on Gram-negative bacteria in combination with colistin [70]. Further, a novel DAP conjugate, which consists of DAP and a mixed ligand analog of the natural* Acinetobacter baumannii (A.baumannii) *selective siderophore, not only retains the inhibitory effect on Gram-positive bacteria but also has potent activity against multidrug-resistant strains of* A.baumannii* [71]. Similarly, several DAP novel conjugates are summarized in Table 3.

There are few drug delivery research reports to improve DAP preparations. In the interest of increasing the effectiveness, DAP-loaded nanoparticles are used directly for treatment of endophthalmitis [72]. Additionally, DAP-modified liposomes exhibit highly specific targeting and therapeutic efficacy against MRSA [73]. More interestingly, an efficient hybrid of inorganic antimicrobial agent with DAP, which is conjugated through a strong covalent bond, enhances bactericidal activity by generating ROS, further damaging cell lipid membranes, and accelerating cell death [74]. In order to increase drug loading, some researches develop a positively charged proliposomal formulation for oral administration of DAP [75]. So, improving preparation of DAP, such as oral preparation, may be a good method to explore the potential of DAP.

## Figures and Tables

**Table 1:** DAP treatment in cancer.

Cancer type	Infections type	Pathogen	Dosage	Ref
Hematologic malignancy Solid tumor	Catheter-related blood stream infections	Gram-positive organisms	6 mg/kg/day i.v.	[44]
Colon cancer	Implantable intra-arterial catheter infections	Not mentioned	10 mg/kg/day i.v.	[45]
Hematologic malignancy Solid tumor	Bacteremia	Vancomycin-resistant Enterococcus	About 6.1 mg/kg/day i.v.	[46]
Acute myeloid leukemia	Bacteremia	Teicoplanin-resistant Gram-positive cocci	8 mg/kg/day i.v.	[16]

**Table 2:** DAP combination therapies against ^a^DNS strains.

DAP combination	Strains	For DNS strains	Mechanisms	Activity	Refs
DAP+Oxacillin	MRSA	^b^Syn	Changes in peptidoglycan insertion Reduction in membrane amounts of ^c^PBP2a	In vivo, In vitro	[58]
DAP+Ceftaroline	MRSA	Syn	Enhance the binding of DAP	In vitro	[61, 62]
DAP+Gentamicin	MRSA	Syn	Not mentioned	In vitro	[66]
DAP+Fosfomycin	VRE	Syn	Reduction in cell surface charge	In vitro	[69]
DAP+Ampicillin	VRE	Syn	Reduction in cell surface charge	In vitro	[59]
DAP+Ceftriaxone	*Streptococcus mitis*	Syn	Prevent DAP resistance emergence	In vitro	[63]

^a^DNS, DAP-nonsusceptible.

^b^Syn, synergy.

^c^PBP2a, penicillin binding protein 2a.

**Table 3:** Novel DAP conjugates.

DAP conjugates	The compounds coupled with DAP	Strains	Mechanisms	Activity	Refs
Synthetic sideromycin	A mixed ligand analog of the natural *A. baumannii* selective siderophore	*A. baumannii*	Actively transport DAP into Gram-negative bacteria	In vitro, In vivo	[71]
Dapt-PEG-DSPE	N-hydroxysuccinimidyl-polyethylene glycol-1,2-distearoyl-sn-glycero-3-phosphoethanolamine	MRSA252	Target on the surface of liposomes	In vitro, In vivo	[73]
AgNCs-DAP hybrid	Silver nanoclusters (AgNCs)	*S. aureus* ATCC 25923	Generate localized high ROS concentration Damage the bacterial membrane and DNA	In vitro	[74]

## Abbreviations

DAP: Daptomycin
MRSA: Methicillin-resistant* Staphylococcus aureus*
VRE: Vancomycin-resistant* Enterococci*
FDA: Food and Drug Administration
MIC: Minimal inhibitory concentration
PG: Phosphatidylglycerol
PBP1: Penicillin binding protein 1
DNS: DAP-nonsusceptible.
